# A large retroperitoneal lipoblastoma as an incidental finding: a case report

**DOI:** 10.1186/s12887-021-02628-w

**Published:** 2021-04-04

**Authors:** Elena Gerhard-Hartmann, Verena Wiegering, Clemens Benoit, Thomas Meyer, Andreas Rosenwald, Katja Maurus, Karen Ernestus

**Affiliations:** 1 Department of Pathology, University of Würzburg, Josef-Schneider-Str. 2, 97080 Würzburg, Germany; 2 Comprehensive Cancer Center Mainfranken, Würzburg, Germany; 3 University Children’s Hospital Würzburg, Würzburg, Germany; 4 Division of Pediatric Radiology, University Department of Radiology, Würzburg, Germany; 5 Division of Pediatric Surgery, University Medical Center ZOM, Würzburg, Germany

**Keywords:** Retroperitoneal tumor, Pediatric, Lipoblastoma, *PLAG1* rearrangement, Case report

## Abstract

**Background:**

Lipoblastoma is a rare benign mesenchymal neoplasm of infancy that most commonly occurs on the extremities and trunk but can arise at variable sites of the body. Retroperitoneal lipoblastomas are particularly rare but can grow to enormous size, and preoperative diagnosis is difficult with diverse, mostly malignant differential diagnoses that would lead to aggressive therapy. Since lipoblastoma is a benign tumor that has an excellent prognosis after resection, correct diagnosis is crucial.

**Case presentation:**

A case of a large retroperitoneal tumor of a 24-month old infant that was clinically suspicious of a malignant tumor is presented. Due to proximity to the right kidney, clinically most probably a nephroblastoma or clear cell sarcoma of the kidney was suspected. Radiological findings were ambiguous. Therefore, the mass was biopsied, and histology revealed an adipocytic lesion. Although mostly composed of mature adipocytes, in view of the age of the patient, the differential diagnosis of a (maturing) lipoblastoma was raised, which was supported by molecular analysis demonstrating a *HAS2-PLAG1* fusion. The tumor was completely resected, and further histopathological workup led to the final diagnosis of a 13 cm large retroperitoneal maturing lipoblastoma. The child recovered promptly from surgery and showed no evidence of recurrence so far.

**Conclusion:**

Although rare, lipoblastoma should be included in the differential diagnoses of retroperitoneal tumors in infants and children, and molecular diagnostic approaches could be a helpful diagnostic adjunct in challenging cases.

## Background

Lipoblastoma is a rare benign mesenchymal neoplasm of infancy and early childhood, occurring typically in children under the age of 3 years, but occasionally in older children and very rarely even in adults [1–3]. In most series, there is a predominance for boys reported [2, 4]. It is a neoplasm of embryonal white fat cells, usually presenting as well-circumscribed tumor localized on the extremities and trunk, but may also occur as a diffuse process (lipoblastomatosis) [1]. However, lipoblastomas may arise at many sites, including the retroperitoneum, the mediastinum and the head and neck region [2, 4, 5], and the clinical differential diagnosis of lipoblastoma, particularly in rare locations, is broad**.** Although benign, lipoblastoma can show local recurrence, especially if incompletely excised [6].

Grossly, lipoblastomas are typically encapsulated with a pale yellow, often lobulated and variably myxoid cut surface. Most tumors measure 3 cm to 5 cm, although very large tumors (up to 28 cm in retroperitoneal localization) are reported [2, 5, 7]. Histologically lipoblastoma is composed of white fat cells at different maturation stages including lipoblasts, immature and mature adipocytes with traversing fibrous septae. In addition, at various proportions, a myxoid change as well as some mesenchymal cells and a plexiform vasculature can be observed [1, 2]. Some lipoblastomas may show a particular prominent myxoid appearance, which, together with the previously described vasculature, leads to morphological similarities to myxoid liposarcoma, which is an important differential diagnosis but is exceptionally rare in this age group [8]. On the other hand, in their large series of 59 lipoblastomas, Coffin et al. reported in 76% an extensive maturation towards mature adipose tissue [2], which may obscure the diagnosis, particularly in small biopsies: the lesion may be misclassified as lipoma or completely missed due to histologic similarity to orthotopic adipose tissue. On the molecular level, lipoblastomas typically show a rearrangement of the chromosomal region 8q11–13, which results most commonly in a fusion of the pleomorphic adenoma gene 1 (*PLAG1*) with diverse partners, most commonly *HAS2* (8q24.1) and *COL1A2* (7q22) [9, 10].

We here report a case of a large retroperitoneal lipoblastoma of a 2-year-old infant that was difficult to diagnose preoperatively, in order to remind that this tumor -albeit very rare in this location- can be an important benign differential diagnosis.

## Case presentation

A 24-month-old girl presented on a routine physical exam with a mass lesion in the right abdomen, which was not tender when palpated. There were no congenital abnormalities, and the development of the child was normal without evidence of any disease so far. The laboratory parameters (including hemoglobin, NSE, alpha-fetoprotein and beta-HCG as well as urine catecholamines) were normal.

**Fig. 1:**
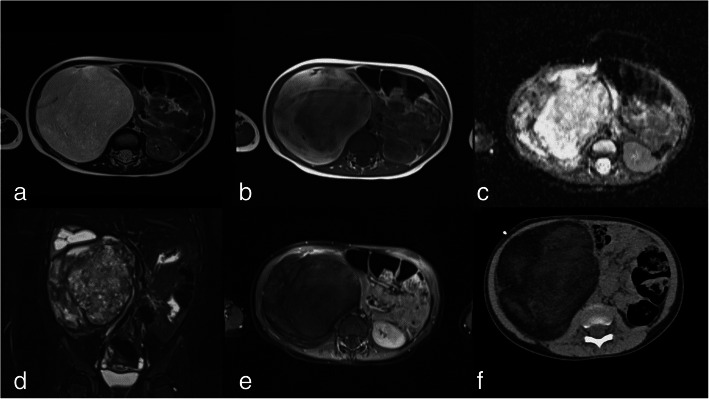
Axial abdominal magnetic resonance imaging (MRI) (**a**-**e**) and computer tomography (CT) (**f**) scan shows a right retroperitoneal mass with similar signal intensity to subcutaneous fat on T2-(**a**) and T1-(**b**) weighted images as well as on the CT scan. There is no diffusion restriction on the diffusion weighted images (**c**) and no significant contrast enhancement (**e**, axial T1-weighted fat suppressed image after intravenous contrast administration). The mass shows a capsule without local infiltration, but cranial displacement of the right kidney with signs of congestion (**d**, coronal fat-saturated T2 –weighted MRI), which gradually resolved after resection (not shown)

Abdominal ultrasound showed a relative homogenous retroperitoneal mass adjacent to the liver and right kidney that measured up to 12.3 cm. MRI revealed a heterogeneous myxoid signal pattern (Fig. 1 a-e). A connection to the right kidney could not be safely excluded. Thus, clinically a malignant tumor, most probably a nephroblastoma or clear cell sarcoma of the kidney, was suspected. In Germany, nephroblastoma would be treated after unambiguous diagnostic imaging according to SIOP2001/GPOH protocol with preoperative chemotherapy without biopsy. However, since the radiological picture was not entirely clear, it was decided to perform a biopsy before systemic treatment. Computer tomography (CT) during biopsy revealed a fat-isodense nature of the mass (Fig. 1 f).

**Fig. 2:**
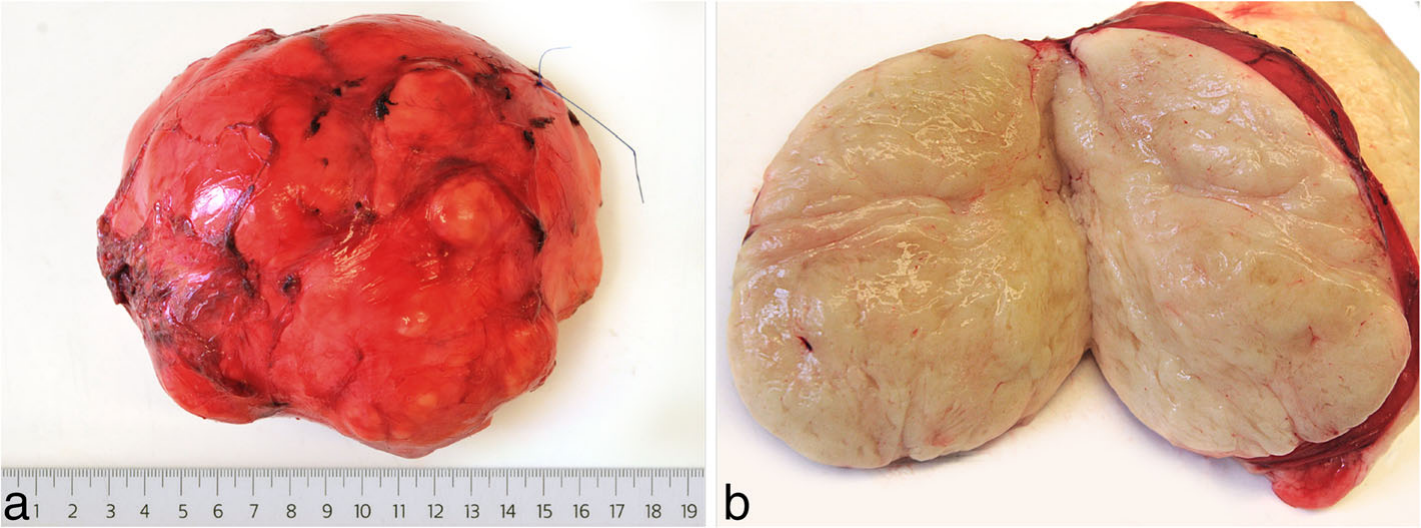
Gross appearance of the resected retroperitoneal mass: The tumor is covered by a thin fibrous capsule (**a**) and shows a pale yellow, slightly lobulated cut surface with small cysts (**b**)

**Fig. 3:**
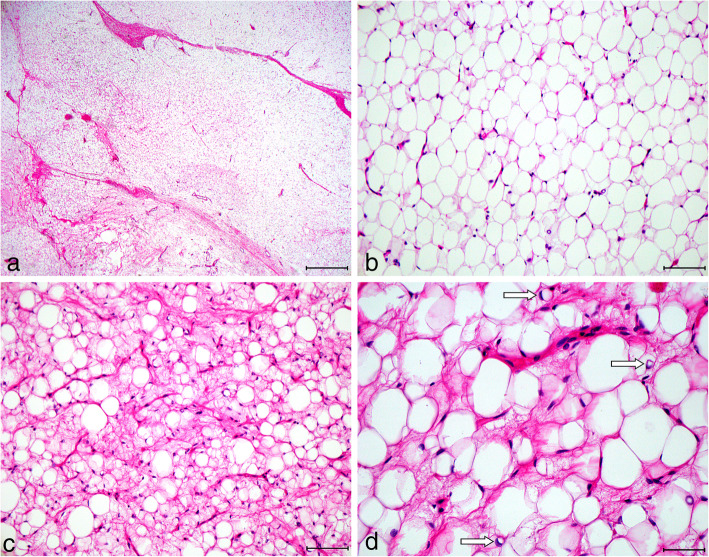
Microscopic examination of the resection specimen revealed a fatty tumor with focal fibrous septae (**a**) and morphologically different areas with focal myxoid appearance and lipoblasts (**c**, **d**), but also areas with much more mature adipocytes (**b**). The arrows in **d** indicate lipoblasts. The length of the scale bar is 500 μm in **a**, 100 μm in **b** and **c**, and 50 μm in **d**

We received fragmented biopsies that are histologically composed of mostly mature adipocytes of slightly variable size with only very focal myxoid stroma, but with some small, slightly curved blood vessels. Although many histologic aspects of the biopsy resemble orthotopic fat tissue, since the tissue reliably stems from the tumor, the diagnosis of a benign lipomatous tumor was rendered, and considering the age of the patient most likely a lipoblastoma was suggested. To corroborate this diagnosis, we performed anchored multiplex PCR based targeted RNA sequencing using the Archer FusionPlex Sarcoma Panel and identified a *HAS2-PLAG1* fusion (*HAS2*: Exon 1, NM_005328.2; *PLAG1*: Exon 3, NM_002655.2). There was no evidence for a rearrangement of the DDIT3 gene and thus no hint for a myxoid liposarcoma. Thus, the diagnosis of lipoblastoma was made, and surgical resection of the tumor was decided. With regard to the surgical approach, median laparotomy versus a right upper abdominal laparotomy was discussed. In order to get a good overview as well safe access to the tumor, the vena cava inferior and the right kidney, it was decided to choose a right upper abdominal laparotomy. After laparotomy, a well-circumscribed mass was encountered in the retroperitoneum that could be completely resected without injury to adjacent structures. Grossly, we found a 13 × 10.5 × 8.7 cm large tumor (weighing 585 g), with a thin, fibrous capsule and a pale yellow, lobulated fatty parenchyma with small cysts (Fig. 2a and b). Histological examination revealed a lipomatous tumor with a vaguely lobular appearance with occasionally fibrous septae (Fig. 3a). The degree of cellular maturation was variable within the tumor, with a focally myxoid appearance and lipoblasts (3c and d), but also areas with much more mature adipocytes (3b), altogether leading to the final diagnosis of a maturing lipoblastoma. The postoperative course was uneventful and the patient recovered promptly from surgery. Follow-up (6 months) revealed no evidence of recurrence so far.

In addition, we performed a literature review by searching the PubMed database using the key words “retroperitoneal lipoblastoma” and “lipoblastoma” and “retroperitoneum” etc. and additional papers were identified by searching the references of relevant articles. We identified 23 cases of circumscribed retroperitoneal lipoblastomas including the here presented case. A tabular overview is given in Table 1, which, however, makes no claim of absolute completeness, since we may have missed single additional reports in journals not published in English or very old reports.

**Table 1:** Reported cases of circumscribed retroperitoneal lipoblastomas (*n* = 23)

Author	Year	Sex	Age	Max. diameter
Tanyel [11]	1986	F	3 years	8 cm
Jimenez [12]	1986	M	12 years	19.5 cm
		M	7 months	15 cm
St Omer [13]	1992	M	5 years	n.r.
Collins [14]	1997	M	2 years 10 months	21 cm
Pollono [15]	1999	M	5 months	14 cm
		F	1 year 7 months	18 cm
Chun [16]	2001	M	2 years 5 months	19.5 cm
Dokucu [17]	2003	M	1 year	12 cm
McVay [18]	2006	M	1 year 5 months	17 cm
De Saint Aubain Somerhausen [3]	2008	F	24 years	> 10 cm
Kok [7]	2010	F	4 years	25 cm
Api [19]	2010	F	22 days	6.2 cm
Burchhardt [20]	2012	F	2 years	15 cm
Susam-Sen [4]	2017	M	1 year	9 cm
		M	2 years 5 months	13 cm
Sakamoto [21]	2018	F	3 years	12 cm
Miyagi [22]	2018	F	3 years	17.5 cm
Abdul-Gafar [5]	2018	F	Not exactly specified, 2–5 years	13 cm
		M	Not exactly specified, 2–5 years	28 cm
Wang [23]	2019	M	1 year 5 months	15 cm
Lopez-Nunez [10]	2020	M	1 year	5.5 cm
Our case	2021	F	2 years	13 cm

*n.r.* Not reported

## Discussion and conclusion

Lipoblastomas are rare benign mesenchymal tumors of infancy and early childhood with often rapid growth that show an excellent prognosis after complete resection. However, the clinical differential diagnosis is broad, particularly in more rarely encountered localizations, and includes various benign and malignant tumors. Retroperitoneal lipoblastoma is especially rare (< 30 well-documented cases, for overview see Table 1), often large and difficult to diagnose preoperatively, and the differential diagnosis in this location comprises primarily malignant tumors including sarcomas, nephroblastomas, neuroblastomas and teratomas.

Histologically, the diagnosis of lipoblastoma can also be challenging, particularly in small biopsies, since lipoblastomas can show morphological variable areas, with a prominent myxoid change, but also regions with an extensive maturation towards mature adipose tissue [2]. The histological differential diagnoses include lipoma, myxoid liposarcoma, well-differentiated liposarcoma/atypical lipomatous tumor and may, particularly in small biopsies of maturing areas, also comprise orthotopic adipose tissue. In the genital area, lipoblastoma-like tumor of the vulva is also among the differential diagnosis [24]. Myxoid liposarcoma and well-differentiated liposarcoma/atypical lipomatous tumor are very rare in the typical age group of patients with lipoblastoma, and show characteristical molecular alterations, namely the translocation t(12;16) (q13;p11) leading to a fusion of the *FUS* and *DDIT3* gene in the former and amplification of the 12q14–15 region affecting *MDM2* and *CDK4* in the latter [1].

Lipoblastoma is characterized on the molecular level by 8q11–13 chromosomal alterations targeting *PLAG1* (pleomorphic adenoma gene 1) located on 8q12 [9]. These alterations lead to PLAG1 overexpression, most commonly caused by chromosomal rearrangements resulting in a replacement of the *PLAG1* promotor by a more active promotor of the fusion partner. The most commonly described *PLAG1* fusion partners are *HAS2* (8q24.1) and *COL1A2* (7q22) [9], but more recently also several other genes (e.g. *COL3A, RAB2A, RAD51L*) are identified to be fused to *PLAG1* in lipoblastoma [10, 25, 26]. Thus, the detection of a *PLAG1* rearrangement, like the classical *HAS2-PLAG1* fusion identified in the presented case, as well as exclusion of the previously mentioned *DDIT3* rearrangement and 12q amplification, nowadays most commonly via FISH and/or targeted RNA sequencing approaches, can be a helpful diagnostic adjunct in challenging cases.

Taken together, Lipoblastomas can occur in a wide variety of localizations with a broad spectrum of clinical differential diagnoses. After complete resection, even patients with very large lipoblastomas have an excellent prognosis. Retroperitoneal lipoblastomas, such as the presented case, are particularly rare but often large tumors, and the clinical differential diagnoses in this setting include highly malignant tumors, like nephroblastoma and clear cell sarcoma of the kidney, that would lead to aggressive therapy. In conclusion, although rare, lipoblastoma should be included in the differential diagnoses of retroperitoneal tumors in infants and children and although the histopathological picture is the mainstay for the correct diagnosis, molecular diagnostic approaches may be a helpful diagnostic adjunct in challenging cases.

## Data Availability

The data and material of this case report are available from the corresponding author upon reasonable request.

## Abbreviations

PLAG1: Pleomorphic adenoma gene 1
NSE: Neuron specific enolase
beta-HCG: Human chorionic gonadotropin
MRI: Magnetic resonance imaging
CT: Computer tomography
FISH: Fluorescence in situ hybridization
